# Aneurysm of an autologous aorta to right coronary artery reverse saphenous vein graft presenting as a mediastinal mass: a case report

**DOI:** 10.1186/1757-1626-1-340

**Published:** 2008-11-20

**Authors:** Thomas M Pulling, Walter Y Uyesugi

**Affiliations:** 1 Department of Radiology, MCHK-DR, Tripler Army Medical Center, 1 Jarrett White Road, Honolulu, HI 96859, USA

## Abstract

Aneurysmal dilation of saphenous vein grafts is a relatively rare complication of the now common surgical procedure of coronary artery bypass graft (CABG) surgery. The true prevalence of this condition is not clear, however, literature review by Jorgensen et. al. between 1975 and 2002 revealed only 76 published cases. [1] Recent review of literature, utilizing OVID (search terms: saphenous vein, aneurysm, graft, pseudoaneurysm, coronary bypass) suggests a significantly higher prevalence with 14 such cases published in a variety of multinational journals during the period of 2006 to April 2007. The causes of this dramatic increase is likely multifactorial, however, in the author's opinion, likely reflects the increased sophistication and utilization of cross sectional imaging modalities. Regardless of the true prevalence of the condition, there is little debate that the potential for serious morbidity and mortality in this patient population is significant, and that increased detection and discussion of viable therapeutic options is critical. [1] Therefore, we present a case report and discussion of a patient with symptomatic cardiac ischemia, found to have a large saphenous vein graft aneurysm (SVGA) on coronary CTA.

## Clinical data

Our patient is a 61 year old male with a history of coronary artery disease (CAD), hypertension (HTN), and hyperlipidemia (HPL) who was successfully treated with 4 vessel CABG in 1997 for symptomatic disease. The patient presented with anginal chest pain to an outside facility in March of 2007 and was found to have a NSTEMI with a troponin peak of 16.7. He was treated medically with integrillin, plavix, aspirin, and lovenox with resolution of symptoms and laboratory abnormalities, and transferred to our center for definitive evaluation and therapy.

The patient reported occasional non-specific chest discomfort and tightness that did not have association with exertion, dyspnea, nausea, vomiting, upper extremity pain, jaw pain, or diaphoresis. Physical examination revealed no significant abnormalities. The 12-lead ECG performed in the clinic revealed borderline right atrial hypertrophy and no evidence of ischemia or prior infarction with a normal sinus rhythm.

## Imaging data

**Figure 1:**
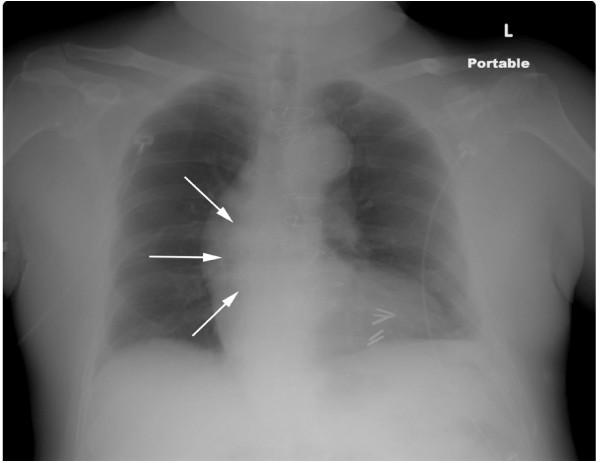
Portable chest radiograph demonstrating a 4.4 cm mass at the right AV groove as a double density (white arrows) of the ascending aorta.

Portable chest radiograph revealed a markedly tortuous thoracic aorta, with a prominent rounded soft tissue double density over the aorta at the right atrio-ventricular junction (Figure 1). There was no evidence of cardiomegaly, pulmonary venous hypertension, pleural effusion, airspace or interstitial disease.

Nuclear myocardial perfusion study utilizing Tc-99 Sestamibi and Bruce protocol graded exercise stress test revealed a region of reversible myocardial ischemia in the lateral wall of the left ventricle with anginal chest pain (subjectively reported at 4/10), 1 mm horizontal ST depressions in leads II, III, AvF, V3, V4, and V5. Exercise was halted at 9:02 secondary to these findings with the patient having reached target heart rate. The ejection fraction was normal at 57%.

**Figure 2:**
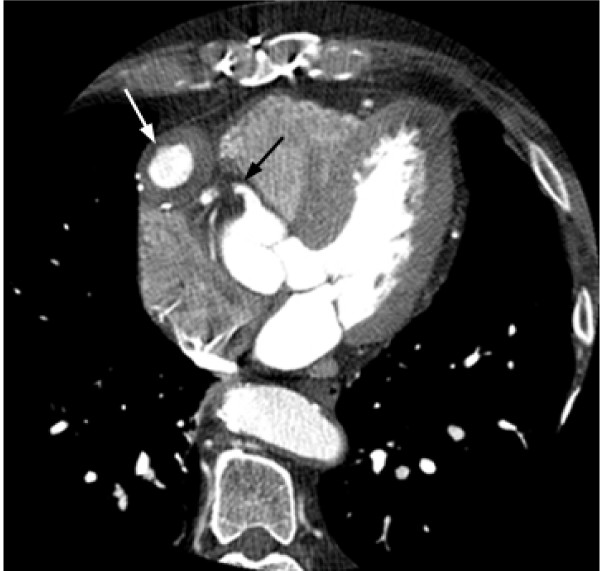
**Axial image from CTA demonstrating a 3.9 cm aneurysm of the proximal aspect of the saphenous vein graft in the right AV groove (white arrow).** Contrast opacification is seen in the lumen with mass effect on the adjacent native RCA (black arrow).

**Figure 3:**
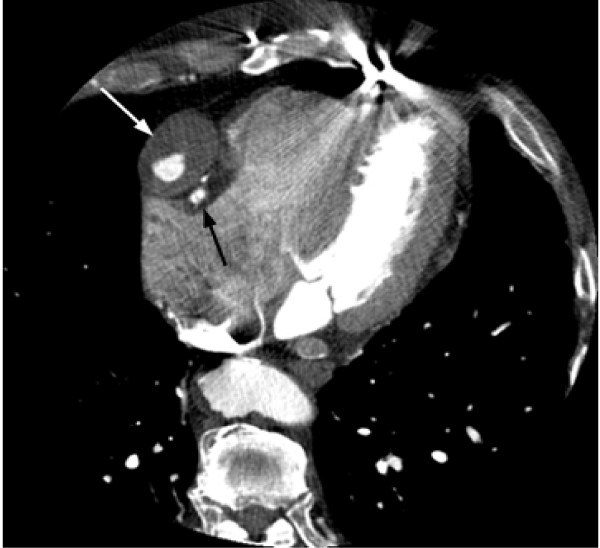
**Axial image from CTA demonstrating a 3.9 cm aneurysm of the proximal aspect of the saphenous vein graft in the right AV groove (white arrow).** Contrast opacification is seen in the lumen with mass effect on the adjacent native RCA (black arrow).

**Figure 4:**
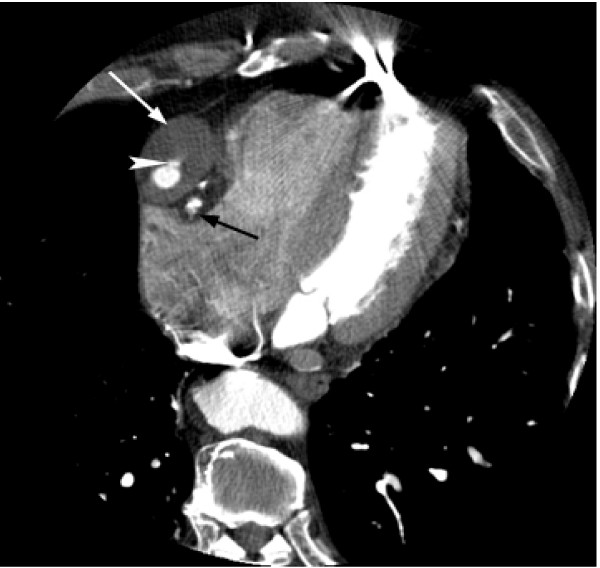
**Axial image from CTA demonstrating a 3.9 cm aneurysm of the proximal aspect of the saphenous vein graft in the right AV groove (white arrow).** Contrast opacification is seen in the lumen with mass effect on the adjacent native RCA (black arrow). Contrast can be seen extending anteriorly within a ulceration in the thrombosed portion of the aneurysm lumen (white arrowhead).

**Figure 5:**
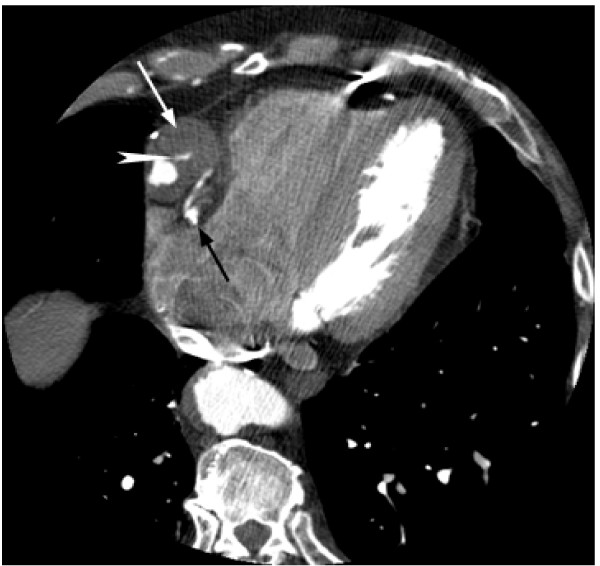
**Axial image from CTA demonstrating a 3.9 cm aneurysm of the proximal aspect of the saphenous vein graft in the right AV groove (white arrow).** Contrast opacification is seen in the lumen with mass effect on the adjacent native RCA (black arrow). Contrast can be seen extending anteriorly within an ulceration in the thrombosed portion of the aneurysm lumen (white arrowhead).

**Figure 6:**
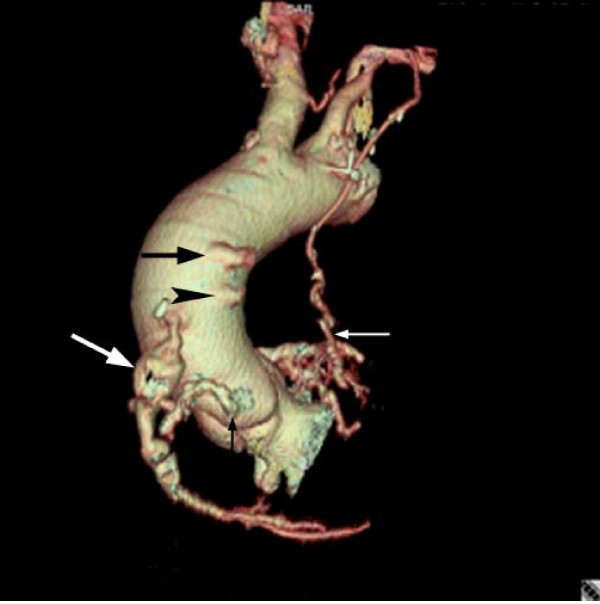
Three dimensional reformatted image from CTA demonstrating the fusiform saphenous vein graft aneurysm (large white arrow) as well as the two thrombosed trunks of the other two saphenous vein grafts (large black arrow and black arrowhead), the native RCA (small black arrow) and the patent LIMA to LAD graft (small white arrow).

Gated CT Coronary angiography was subsequently performed utilizing a GE 16 slice MDCT and approximately 125 cc of Visipaque 320. Test conditions were optimal with a normal sinus rhythm and heart rate between 54 and 55 bpm. A 3.9 cm fusiform aneurysm of an anterior aorta to RCA reverse saphenous vein graft was identified just distal to the origin of the native RCA with severe proximal narrowing of the graft (3.5 mm luminal diameter), but patent contrast opacification through the distal RCA (Figure 2, 3, 4, 5, and 6). The aneurysm wall is dominated by laminated appearing thrombus, comprising greater than fifty percent of the diameter (largest luminal diameter 1.6 cm). Significant mass effect with lateral and posterior displacement of the native RCA was identified. Thrombosed SVG's were seen from the anterior aorta to the second diagonal branch of the LAD and the second obtuse marginal branch of the LCA. A patent LIMA to LAD graft with anastamosis distal to the third diagonal branch was seen.

**Figure 7:**
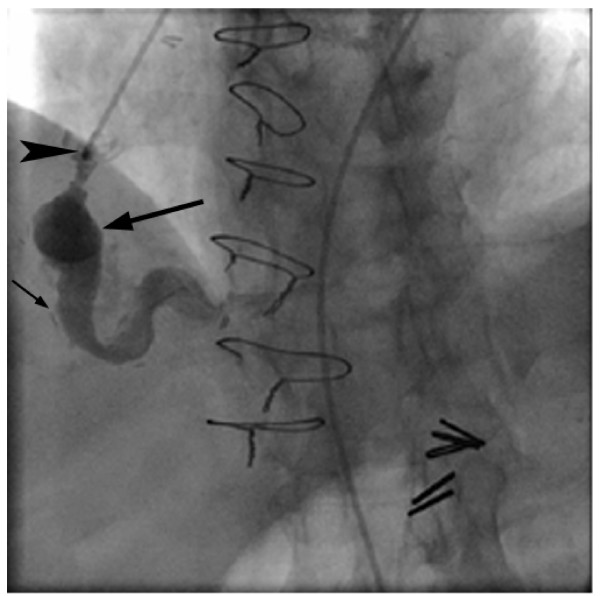
**Selective injection of the aorta to RCA reverse saphenous vein graft.** The tip of the catheter is seen at a mildly narrowed ostium (arrowhead), and the lumen of the saphenous vein graft aneurysm (large arrow) with areas of ulceration (small arrow) reproduce the findings seen previously at coronary CTA.

Coronary angiogram was subsequently performed and correlated well with the coronary CTA performed prior. Moderate proximal stenosis was identified at the origin of the reverse saphenous vein graft to RCA, with a long mid segment fusiform aneurysm with two segments of more prominent luminal dilation (figure 7). Also identified, and consistent with coronary CTA, was occlusion of the grafts to the circumflex and LAD with a diseased, but patent LIMA to LAD graft.

A multidisciplinary conference composed of cardio-thoracic surgeons, cardiologists, nuclear medicine physicians, and radiologists was then convened to discuss the data and treatment options. In accordance with what we believe practice guidelines dictate, and upon the wishes of the patient, our patient was released from the hospital symptom free with close clinical follow-up.

## Discussion

Saphenous vein graft aneurysm (SVGA) is an uncommon complication of coronary artery bypass graft (CABG) surgery with potentially lethal complications. [2] Complicating this potentially fatal complication is the frequently asymptomatic nature of these lesions, with at least 59% of the detected lesions found incidentally in asymptomatic patients. [3] In addition to the obvious risk of sudden death from rupture, a myriad of complications have been described to include thrombotic embolus with acute coronary syndrome (ACS), fistula formation with hemoptysis, graft occlusion with ACS, and compression of adjacent vital structures producing ACS or superior vena cava syndrome. [4]

Saphenous vein graft aneurysms, like aneurysms in any vessel, can be categorized as true aneurysms and pseudoaneurysms. The major distinction in this regard being that the true aneurysm involves dilation of the vessel in a fashion in which all three histologic tissue layers of the vessel wall are intact. [1] In the case presented, the aneurysm is fusiform in shape, involves the mid portion of the vessel, and presented approximately ten years following CABG in a man with clear risk factors for atherosclerosis. This is the typical pattern for a true aneurysm of a saphenous vein graft. [1] Psuedoaneurysms are typically saccular, occur early in the post-operative period, and are near the sites of anastamosis. [1] The significance of this distinction is not purely academic as pseudoaneurysms may lend themselves to more successful treatment utilizing minimally invasive procedures such as covered stent placement, [5] or coil embolization. [6] In contradistinction, true fusiform aneurysms will typically require a surgical approach for correction, [6] as was the case with our patient in which intervention at coronary angiography could not be attempted.

A variety of factors have been implicated as potential contributing factors in the development of both true and pseudoaneurysms of saphenous vein grafts. These include post operative infection, tight suture anastomoses, suture dehiscence, trauma to the vessel during harvest, intrinsic weakness in venous walls at sights of branching or valve attachment, grafting of varicose veins, steroid therapy, as well as the typical atherosclerotic disease risk factors, ie. hyperlipidemia, smoking, hypertension etc. [1] Clearly the type of aneurysm that develops, true aneurysm versus pseudoaneurysm, is more likely with certain of these contributing factors.

Given the rather dramatic increase in published reports of SVGA in the recent literature (see introduction), one must question the etiology. There has been a trend toward increasing utilization of endoscopic saphenous vein harvest versus traditional open saphenous vein harvest with improved patient outcomes with respect to the harvest site. [7] An intriguing possibility centers around the theoretical potential for increased trauma and thus poorer quality saphenous vein grafts with greater likelihood for development of aneurysms with endoscopic saphenous vein harvest. [7] This is particularly intriguing as a potential cause of a true increase in the incidence of saphenous vein graft aneurysms when the experience of the surgeon with the technique is limited. [7] Several authors have reported a similar patency of these endoscopically harvested grafts in comparison to traditional open harvest techniques, [8] however, to our knowledge, no study looking at the prevalence of saphenous vein graft aneurysms in these two populations has been reported. It should be stated that most cardiothoracic surgeons feel that the quality of endoscopically harvested grafts are equal that of open harvested grafts. [7] However, alterations in surgical technique in association with a potential true increase in the incidence of SVGA, warrants a retrospective study of patients with saphenous vein graft aneurysms, focusing attention to surgical technique.

Additional considerations include the increased utilization of coronary artery bypass graft surgery in the treatment of coronary artery disease in the United States. This theoretically would lead to an increase in the prevalence of SVGA, and thus an increase in the reported prevalence of the condition without altering the percentage of patients suffering this complication. While this is an attractive conclusion, one must also consider the tempering effect of this trend created by increased utilization of coronary angioplasty and stenting as primary modes of therapy for symptomatic coronary artery disease.

While these avenues of thought are intriguing, perhaps the most likely reason for the increased prevalence of this condition in the literature is an increase in the rate of detection of this often asymptomatic condition [3] as a result of the profound increased utilization and sophistication of cross sectional imaging, particularly computed tomography, in the evaluation of a variety of suspected clinical conditions. [9] Refined techniques such as ECG gating and technology advancements such as multi-detector CT (MDCT) have allowed for high quality diagnostic images of native and graft coronary anatomy to be obtained noninvasively. [10] The additional benefits of CT compared with coronary angiography alone, such as multiplanar reconstructions, 3-Dimensional reconstructions, visualization of the entire vessel as opposed to the lumen exclusively, and visualization of surrounding anatomy for surgical planning have led to an increase in the use of coronary CTA in the evaluation and management of patients with suspected coronary artery disease. [11] Traditional catheter coronary angiography will clearly detect most native and graft coronary aneurysms. A striking limitation of this technique, however, is visualization of only the lumen of the vessel. Case reports in the literature demonstrate the advantage of coronary CT in visualization of the entire vessel, with the ability to detect aneurysmal dilation of the vessel when traditional coronary angiography cannot. [12] This exponential increase in the utilization of CT, and to a lesser extent other imaging modalities, undoubtedly has led to an increase in the reported cases of SVGA.

Treatment for SVGA remains a difficult question, and certainly depends upon multiple critical clinical parameters. As we have seen, some SVGA may be successfully treated percutaneously with methods such as coiling [6] or covered stents, [5] however, percutaneous treatment is not without problems, even in seemingly appropriate clinical settings. Part of the difficulty in defining optimal treatment is the rarity of the condition, combined with the frequently asymptomatic nature of the condition, and the potential morbidity and or mortality associated with the treatment options. Further complicating the matter is the case report and case series nature of the available data upon which to base treatment decisions. In the largest case series, reporting outcome data in thirteen patients, Dieter et. al. demonstrated no significant survival difference in retrospective review of patients treated conservatively with medical management compared to those treated with surgical therapy for SVGA. [13] Clearly more data is required to clearly define optimal treatment in this patient population.

## Conclusion

SVGA is a relatively uncommon complication of CABG surgery, with potentially lethal consequences. [1] Similar to aneurysms in any location, these aneurysms may be true or pseudoaneurysms, [1,6] with specific characteristics and risks factors for each, and different therapeutic options. [1,5,6] The rather dramatic increase in published reports of this condition certainly suggests that we are diagnosing this often asymptomatic condition as a result of increased utilization of imaging for a variety of idications. [9] Other intriguing possibilities include an increase in the true incidence of this condition as a result of factors such as alteration in surgical technique. Treatment remains an area of considerable uncertainty with available data based on case reports and small case series. As sophisticated cross-sectional imaging and surgical treatment of CAD are both becoming more frequent, we are likely to encounter SVGA more frequently, which is borne out in recent literature search. Our task will be to continue to gather relevant data, considering multiple etiologies, with multi-disciplinary reviews in order to arrive at a clear understanding of why this is occurring, and define the optimal approach to management.

## Consent

All imaging and invasive procedures performed in the acquisition of the data presented in this document were obtained after obtaining written informed consent from the patient according to the policies of the authors' institution. Furthermore, the final document removes all specific patient identifying information, and was approved for publication by the home institution publication review process. All consent documents and institutional review documents are available for review by the Editor-in-Chief of this journal.

## Competing interests

The authors declare that they have no competing interests.

## Authors' contributions

TMP participated in the interpretation of initial coronary CTA imaging, performed literature search, participated in multidisciplinary conference, completed original manuscript and revisions, and formatted images for publication. WYU participated in protocol of coronary CTA imaging, initial interpretation of coronary CTA imaging, literature search, multidisciplinary conference, proofing of manuscript and revisions, and image selection and technical support in formatting of images for publication.
